# The relationship between immuno-biochemical indicators and clinical manifestations of alcoholic and/or mixed forms of addiction in adolescents

**DOI:** 10.1192/j.eurpsy.2025.939

**Published:** 2025-08-26

**Authors:** T. V. Chernobrovkina, S. A. Zozulya, Z. V. Sarmanova, I. N. Otman, A. V. Masyakin, E. V. Bryun, I. Y. Kotova

**Affiliations:** 1 FGBUZ “Moscow Scientific and Practical Center of Narcology”, DZM; 2 FSBSI “Mental Health Research Centre”, Moscow, Russian Federation

## Abstract

**Introduction:**

Identification of the effects of experimentation with psychoactive substances and the formation of addictive behavior in adolescents in risk groups requires the development of new approaches to the clinical and laboratory examination for the prevent severe complications of intoxication and treatment of addiction. One of the biological indicators of complications from the use of psychoactive substances during the period of brain development may be neuroinflammation, in addition to metabolic disorders and disorders of other internal organs.

**Objectives:**

To study the relationship between the levels of inflammatory markers and biochemical blood indicators with the clinical symptoms in adolescents with alcohol and substance intoxication.

**Methods:**

Clinical and laboratory examinations included 40 patients aged 14 to 17 years diagnosed with behavioral and mental disorders due to alcohol use and/or combined alcohol and substance abuse. In blood plasma, the activity of leukocyte elastase (LE) and α1-proteinase inhibitor (α1-PI), as well as the level of autoantibodies (AB) to S100B and basic myelin protein (MBP) were measured. The results were compared with the corresponding normative indicators.

**Results:**

The relationship between the level of immune system activation and the activity of the pathological process in the brain served as the division of patients into groups. The 1st group (58.3%) was characterized by high activity of LE and α1-PI and an increased level of aAT to MBP (p<0.05). The 2nd group (42.7%) was distinguished by low LE activity and a high level of other markers (p<0.01). In the 1st group, a higher monocyte content, an increase in creatine phosphokinase activity, uric acid level, aspartate aminotransferase and alanine aminotransferase ratio, a decrease in gamma-glutamyltransferase activity and serum iron and ferritin level were found compared to the 2nd group. In the 2nd group, the increase in the number of neutrophils was associated with a relatively increased platelet content, as well as higher levels of alkaline phosphatase activity, creatinine content, total and direct bilirubin.

In the 1st group, signs of attention deficit, autoaggression, and increased tolerance to the dose of the substance used were detected more often. In the 2nd group, pronounced tension and irritability, a longer duration of drug use, and more severe clinical manifestations of withdrawal syndrome were observed.

**Conclusions:**

The introduction of indicators of neuroinflammation, associated with ferroptosis mechanisms, initially clinically asymptomatic, into the cluster of clinical and laboratory studies will specify the diagnostics of individual changes in reactivity and health disorders in adolescents at the stage of drug addiction. This will substantiate and increase the effectiveness of the prevention of addictions and early disability among adolescents at risk.

**Disclosure of Interest:**

None Declared

